# Artificial Intelligence and its future potential in lung cancer screening

**DOI:** 10.17179/excli2020-3095

**Published:** 2020-12-11

**Authors:** Christopher Joy Mathew, Ashwini Maria David, Chris Mariya Joy Mathew

**Affiliations:** 1 Acute Medicine Department, Conquest Hospital, East Sussex Healthcare NHS Trust, United Kingdom; 2 Jubilee Mission Medical College and Research Institute, Kerala University of Health Sciences, Kerala, India; 3 David Tvildiani Medical University, Tbilisi, Georgia

**Keywords:** artificial intelligence, lung neoplasms, detection of cancer, machine learning, lung cancer screening, artificial intelligence and lung cancer, low-dose computed tomography, artificial intelligence in radiology, computer-aided diagnosis, convolutional neural networks (CNN)

## Abstract

Artificial intelligence (AI) simulates intelligent behavior as well as critical thinking comparable to a human being and can be used to analyze and interpret complex medical data. The application of AI in imaging diagnostics reduces the burden of radiologists and increases the sensitivity of lung cancer screening so that the morbidity and mortality associated with lung cancer can be decreased. In this article, we have tried to evaluate the role of artificial intelligence in lung cancer screening, as well as the future potential and efficiency of AI in the classification of nodules. The relevant studies between 2010-2020 were selected from the PubMed database after excluding animal studies and were analyzed for the contribution of AI. Techniques such as deep learning and machine learning allow automatic characterization and classification of nodules with high precision and promise an advanced lung cancer screening method in the future. Even though several combination models with high performance have been proposed, an effectively validated model for routine use still needs to be improvised. Combining the performance of artificial intelligence with a radiologist's expertise offers a successful outcome with higher accuracy. Thus, we can conclude that higher sensitivity, specificity, and accuracy of lung cancer screening and classification of nodules is possible through the integration of artificial intelligence and radiology. The validation of models and further research is to be carried out to determine the feasibility of this integration.

## Introduction & Background

Artificial intelligence (AI), the term coined by John McCarthy in 1956, can be described as the approach of using computers and technology to simulate intelligent behavior as well as critical thinking comparable to a human being (Amisha et al., 2019[2]). It also enables us to analyze and interpret complex medical data, thus, aiding in the diagnosis, management, and predicting treatment outcome in different clinical presentations. Artificial intelligence has the potential to bring about radical changes in the field of medicine. Obtaining digitized data, machine learning, and computing infrastructure has made AI applications to expand into areas that were previously believed to be impossible without human expertise with its latest advancements (Yu et al., 2018[43]). Deep learning, an AI technique, has swiftly improvised the fields of caption generation, image recognition, and speech recognition in the past few years (Tang et al., 2018[40]). According to Tang, a prime target for early incorporation of such AI techniques in the medical sphere is radiology. The depth, quality, and value of the radiological contribution to quality care of patients and health of the population is expected to be enhanced considerably by the application of AI over the next decade. The workflow of a radiologist is anticipated to make far-reaching progress and transformation.

Among the cancer-related deaths worldwide, lung cancer is the most frequent cause (Hirsch et al., 2017[12]). Lung cancer is diagnosed in 1·8 million people each year, and 1·6 million people die per year due to the disease (Hirsch et al., 2017[12]). According to data provided by the Cancer Research UK, updated in 2020, the net five-year or more survival rate is 13.8 % (Broggio and John, 2019[4]). Worldwide attention is to be directed towards the control of lung cancer. The tertiary prevention of lung cancer has been influenced by the studies on its relative risk factors and the epidemiologic characteristics of lung cancer (Mao et al., 2016[25]). These studies have also explored new ways of diagnosis and treatment of lung cancer. The solitary pulmonary nodules which are mostly benign, present a diagnostic challenge for clinicians since a significant proportion of these can be early lung cancers which are potentially curable (Cruickshank et al., 2019[8]). Seventy-five percent of lung cancer cases are detected only in its advanced stages with nodal spread and metastatic disease, due to the minimal or absence of symptoms in the initial stages of the disease (Murphy et al., 2018[26]). This can influence the survival rate among patients diagnosed with lung cancer. The National Lung Screening Trial (NLST) findings implicated that screening for lung cancer in high-risk individuals using low-dose computed tomography can reduce lung cancer mortality by 20 % (Tammemägi, 2015[39]).

Lung cancer associated with high mortality and morbidity demands improvement and modification of lung cancer screening techniques. While it is not practically possible to screen all the individuals, at least those at higher risk should be screened for lung cancer. Strategies such as chest radiography and sputum cytology for screening have been focussed for several years to reduce deaths due to lung cancer (American Lung Association, 2015[1]). In developing countries where lung cancer has reached epidemic proportions and is still the main cause of cancer-related death, cure rates could be improved by CT screening to routinely detect potentially malignant lung nodules of a few millimeters size in the high-risk populations (International Early Lung Cancer Action Program Investigators et al., 2006[16]; Field et al., 2012[9]). The poor prognosis on late diagnosis and the change of outcome depending on the time of detection necessitates the utilization of advanced technologies in the lung cancer diagnosis and management. Radiologists have spent infinite working hours evaluating, characterizing, and detecting pulmonary nodules through CT lung screening techniques (Rubin, 2015[33]). This points to the need for technological advancements that can help radiologists to decrease the burden and increase the sensitivity of lung cancer screening while maintaining low false-positive rates.

The morbidity and mortality associated with lung neoplasms and other lung conditions need to be reduced by improving the screening for lung conditions and thereby increasing the rates of early detection and appropriate management. The role of machine learning and its advantages on medical imaging diagnosis is to be explored for its future potential in cancer detection. In this article, the impact of artificial intelligence in imaging diagnostics will be reviewed, and evidence for the potential of AI in early detection and management of lung cancer will be explored. Great power is demonstrated in this rapidly growing field of computing medical imaging but with several challenges that have to be addressed before its application into clinical routine. The collaboration between AI and different fields in medicine may increase the interpretability and confidence in AI.

## Methodology

A comprehensive search was conducted in the PubMed database, and based on the relevancy and details, articles were selected from all the currently available literature in the database. No restrictions were imposed based on the age of participants, geographical location, or language. The studies between 2010 to 2020 were included, and animal studies have been excluded. From the primary search results, 39 articles were selected for the review. The data from these articles have been assessed and discussed in this review.

## Review

### Future of artificial intelligence in lung neoplasms

Theoretical physicist Stephen Hawking quoted that within the next 100 years humans will be overtaken by computers with AI and when that happens, the goals of the computers should be aligned with humans. Increasing attention is to be paid to the utility of artificial intelligence in medicine. A E Nikolaev et al. in 2019 discussed the possibilities of machine learning in the chest computed tomography. Even though the amount of information that a radiologist has to process is snowballing due to the use of computed tomography for lung screening, the automatic image analysis makes it possible for more studies to be interpreted (Nikolaev et al., 2019[28]). Nikolaev felt that most software developers operationalize radiology since it is a medical specialty with high digitalization. Researchers from Google AI used a deep learning model for screening the CT images and detecting lung cancer (Jacobs and van Ginneken, 2019[17]). Their results were comparable or better than the performance of radiologists and are promising. However, to implement this, the adjustment of screening guidelines to accept recommendations from black-box proprietary AI systems is required. Kan Chen et al. proposed a new active model based on the fuzzy speed function for the segmentation of pulmonary nodules which are a potential manifestation of lung cancer (Chen et al., 2014[6]). They found that the problem of boundary leakage in segmentation by the classical active contour models (ACM) can be overcome by the fuzzy speed function-based models. Accurate segmentation of pulmonary nodules could be obtained by incorporation of fuzzy speed function into ACM because the fuzzy speed function approaches zero at the boundaries and evolution of contour curve stops. The results also showed that the segmentation of juxta-vascular nodules and GGO nodules which is an active area can be accurately performed with the proposed ACM. Yanli Lin et al. showed for the first time that more accurate identification of lung cancer among indeterminate pulmonary nodules is possible by the integration of plasma biomarkers and radiological characteristics which in the future allows effective treatment for lung cancer while sparing individuals from harmful diagnostic procedures for benign lesions (Lin et al., 2017[22]). Luis M Seijo et al. discusses the possibility of a highly efficient long term outcome by combining deep extraction of data provided by ongoing screening efforts with new mathematical and artificial intelligence techniques (Seijo et al., 2019[35]).

Artificial intelligence techniques such as machine learning and deep learning promise the ability for efficient analysis of the vast amount of data pertaining to the lung conditions. The automated analysis allows improved characterization of pulmonary nodules as well as earlier detection of lung malignancies. But standardization of the radiological image data is required for the clinical and widespread application of computer-based automated studies so that more examinations can be interpreted. Certain models like the Google AI demands further validation. The proposed new model incorporating the fuzzy speed function in a study was found to be superior to the local region information-based ACM in terms of accuracy. With the utilization of artificial intelligence, multivariate logistic regression analysis of the molecular and radiological characteristics is possible and malignant lesions can be distinguished from the benign lesions accurately. It can be very powerful in avoiding the overdiagnosis of lung cancer due to the increased number of indeterminate pulmonary nodules on low dose CT. AI technologies used to select the most suitable combinations of biomarkers such as molecular biomarkers or image-based biomarkers points to the future revolutionary changes in the field of radiology. Even though algorithms will replace the tasks earlier limited to human beings, doctors will always be essential in clinical medicine. Artificial intelligence is expected to increase the precision of medical care by improving disease prediction, diagnosis, treatment, and outcome.

### Artificial intelligence in lung cancer screening

Early detection of cancer plays a significant role in the course of all forms of cancer. Quickly detective, non-invasive, and a high performing method must be available ideally to screen early stages of lung cancer (Huang et al., 2020[14]). Artificial intelligence allows radiologists to efficiently manage the data load of lung cancer screening using computed tomography (Randerath et al., 2020[31]). Baldwin et al. in 2020, conducted a validation study by retrospectively collecting the database of pulmonary nodules of size 5-15 mm which were noted incidentally from three hospitals in the UK. In his study, an AI algorithm, the lung cancer prediction convolutional neural network (LCP-CNN), was compared with the Brock University model, advised in UK guidelines. It was noted that artificial intelligence improves risk prediction, as 24.5 % of nodules scored below the lowest cancer nodule score with LCP-CNN, while 10.9 % of nodules scored below with Brock score (Baldwin et al., 2020[3]). Gray Level Co-event Matrix (GLCM) technique was employed by Reddy et al. for the prediction of lung tumors and when compared with the histogram was found to be more precise in predicting the tumor (Reddy et al., 2019[32]). With a standard probability structure, this framework can effectively determine the tumor position and thus promising a positive outcome for identifying the disease. Chassagnon et al. in his study, emphasized the need for radiologists to utilize technological advancements like AI in the field of chest CT for large-scale cancer screening and lead the way for the latest changes in radiology (Chassagnon et al., 2020[5]). Nasrullah et al. proposed that a deep learning model using customized mixed link network (CMixNet) architectures, combined with clinical factors for the detection of nodules can reduce the false-positive rates and misdiagnosis in the early stages of lung cancer (Nasrullah et al., 2019[27]). It was found to have a higher sensitivity and specificity. Bethany Geary et al. developed an 11 protein marker panel using Sequential window acquisition of all theoretical fragment ion spectra (SWATH) mass spectrometry (Geary et al., 2019[10]). Further development is required for the proteome signature for lung cancer developed by combining a machine learning model with the SWATH mass spectrometry data. It has a 12 protein panel with a mean area under the curve (AUC) and an accuracy of 0.89 each. Shulong Li et al. proposed an algorithm for predicting lung nodule malignancies with higher sensitivity and specificity, by fusing handcrafted features (HF) with the features derived from a three dimensional (3D) deep Convolutional Neural Network (CNN) (Li et al., 2019[21]). This fusion algorithm overcame the disadvantage of HF and compared to the other competitive classification models, it was found to have the highest AUC, sensitivity, specificity, and accuracy. Wenkai Huang et al. developed a fused neural network framework called the Amalgamated-Convolutional Neural Network (A-CNN) and analyzed the performance using Lung Nodule Analysis 16 (LUNA) and Ali Tianchi dataset (Huang et al., 2019[15]). With A-CNN, high sensitivity of 81.7 % and 85.1 % per scan was achieved with the average number of false positives per scan as 0.125 and 0.25 respectively. An *in vitro* cell model was constructed by Jing Song et al. to mimic the epithelial-mesenchymal transition (EMT) in patients and he found that in the early stages of lung adenocarcinoma, three early EMT hallmark genes, GALNT6, SPARC, and HES7 were specifically up-regulated (Song et al., 2019[37]). Moritz Schwyzer et al. assessed the detection of lung cancer by artificial neural networks based on different doses of PET (Schwyzer et al., 2018[34]). At standard-dose PET, the artificial neural network (ANN) has a sensitivity of 95.9 % and specificity of 98.1 %, whereas, ultralow dose PET (3.3 %), has a sensitivity of 91.5 % and a specificity of 94.2 %. Naji Khosravan et al. developed a collaborative computer-aided diagnosis (C-CAD) unifying eye-tracking systems and CAD in realistic radiology room settings (Khosravan et al., 2019[19]). Promising results were obtained while testing the proposed C-CAD system in a lung cancer screening by reading low dose chest CTs by several radiologists. Gregory R Hart et al. trained and validated a multi-parameterized artificial neural network (ANN) for the prediction of lung cancer risk (Hart et al., 2018[11]). The ANN was based on personal health information and provided a non-invasive and cost-effective risk stratification tool with high specificity and modest sensitivity. Ahmed Shaffie et al. proposed a novel system integrating appearance features and geometric features, which showed a nodule classification accuracy of 91.20 %, promising a valuable tool for the detection of lung cancer (Shaffie et al., 2018[36]). Chi-Hsiang Huang et al. tried to combine a chemical sensor array and a machine learning technique so that a breath test can be developed for the detection of lung cancer (Huang et al., 2018[13]). The areas under the receiver operating characteristic curve with linear discriminant analysis and the supportive vector machine technique were 0.91 (95 % confidence interval (CI) = 0.79-1.00) and 0.90 (95 % CI = 0.80-0.99) respectively, which shows the higher accuracy. In an integrated microarray analysis by Wan-Ting Liu et al., CENPA, CDK, and CDC20 genes were seen as a novel cluster of prognostic biomarkers and a technically simple method of lung adenocarcinoma detection (Liu et al., 2018[24]). For the early detection of lung cancer, Wookjin Choi et al. developed a radiomics prediction model for pulmonary nodules with low dose CT (Choi et al., 2018[7]). This model with two CT radiomic features possessed 84.6 % accuracy, which was higher than Lung CT Screening Reporting and Data System (Lung-RADS). Table 1(Tab. 1) (References in Table 1: Baldwin et al., 2019[3]; Choi et al., 2018[7]; Geary et al., 2019[10]; Hart et al., 2018[11]; Huang et al., 2018[13]; Huang et al., 2019[15]; Khosravan et al., 2019[19]; Li et al., 2019[21]; Liu et al., 2018[24]; Nasrullah et al., 2019[27]; Reddy et al., 2019[32]; Schwyzer et al., 2018[34]; Shaffie et al., 2018[36]; Song et al., 2019[37]) summarizes the proposed models for detection of lung cancer in various studies. 

**Figure 1:**
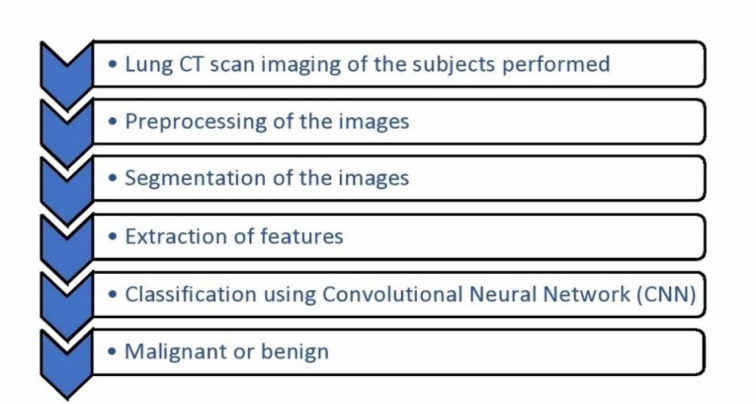
Steps in a deep learning model of lung nodule detection Legend: CT- Computerized Tomography; CNN- Convolutional Neural Network

Artificial Intelligence consistently proves to be a promising advancement. Almost all the studies concluded that the incorporation of AI into the field of radiology will promote improved patient care by earlier and accurate detection of the disease, and thereby a good prognosis. Due to the better discrimination and evaluation of a greater proportion of lung nodules, cancers being missed can be reduced. The development of various artificial intelligence algorithms benefitted thoracic imaging for various conditions. The performance of artificial intelligence can be equivalent to or greater than a radiologist's performance, but a more practical alternative is assisting the radiologists by AI algorithms so that a more efficient system develops. The lack of prospective clinical validation of AI algorithms has to be looked into and adequate validation measures are to be undertaken in the future so that it can be utilized in the routine clinical practice. The novel models of Convolutional Neural Network (CNN) have shown a greater precision in the prediction of tumors compared to a histogram. Figure 1(Fig. 1) depicts the steps in a CNN deep learning model to detect lung nodules. Algorithms developed by fusion of features helped in overcoming the drawbacks or disadvantages of one feature by the advantages of the other and in achieving increased sensitivity, specificity, and accuracy. This was seen in the example of intrinsic CNN features reflecting the unique characteristics of a particular lesion which was not achievable with handcrafted features.

Based on the complementary of HF, it also alleviated the requirement of a large scale annotated dataset for the CNNs. The newly formulated frameworks such as the A-CNN model improvised the efficiency of CT radiological analysis. Several deep learning models have been proposed including those combining clinical features and resulted in a reduction of false positives and misdiagnosis of lung cancer even in the early stages. New applications of fluorodeoxyglucose (FDG)-PET can improve the specificity of lung cancer screening efforts by further improvement of artificial neural network technology. Lower doses of PET can be utilized in the detection of lung cancer. The combination of the machine learning model with spectrometry data enabled the development of highly accurate protein marker panels for lung cancer. Artificial intelligence also made it possible to screen for lung cancer by focusing on the hallmark genes and molecular changes. Newer non-invasive tests such as breath tests are expected to be available following proper validation. Computer-aided diagnosis involving public health information, appearance features, and geometric features promises greater accuracy. Artificial intelligence, including machine learning algorithms, thus put forward the possibility of fully automated lung cancer detection, clinical diagnosis, and drug screening.

### Artificial intelligence in pulmonary nodule classification

Classification and characterization of the stage of the disease are as important as its diagnosis. It plays a pivotal role in the formulation of its management plan. Prayer et al. in his review article mentioned the significant impact of machine learning in computer-aided detection and further characterization of pulmonary nodules proving to be a valuable resource in lung cancer screening programs (Prayer et al., 2020[30]). Onishi et al. performed an analytical study for the classification of pulmonary nodules based on a deep convolutional neural network (DCNN) and generative adversarial networks (GAN) (Onishi et al., 2020[29]). Sixty patients with a confirmed diagnosis on biopsy were analyzed and evaluated with the help of CT scans which yielded a specificity of 77.8 % and a sensitivity of 93.9 %. The study also showed the improvement of classification accuracy with the use of GAN- generated images, compared to data augmentation, even for medical datasets that have fewer images. Another method was proposed based on a combination of Fuzzy C-means (FCM) clustering and classification learning, for the classification of pulmonary nodules (Liu et al., 2015[23]). The study revealed that more precise segregation of ground-glass opacity (GGO) pulmonary nodule, vascular, and pleural adhesion can be obtained from this proposed method than the other typical algorithms. Li et al. studied 346 healthy people (male: female = 221:125, mean age 51 years) with pulmonary nodules, from a lung cancer screening program conducted between 2017 March to November with the help of a deep learning-based computer-aided diagnosis (DL-CAD) system and double reading independently, and their performance in nodule detection and characterization were evaluated by combining the results (Li et al., 2019[20]). The result showed a higher detection rate with DL-CAD system (86.2 %) than double reading (79.2 %), regardless of nodule size (P < 0.001); if nodules ≥ 5 mm, detection rate with DL-CAD is 96.5 % and double reading is 88.0 % (P = 0.008); and for nodules < 5 mm, 84.3 % and 77.5 % for DL-CAD and double reading respectively with P < 0.001. Shu-Ju Tu et al. integrated localized thin-section CT with machine learning classification and radiomics features extraction in 2018 which was then supervised by histological diagnosis (Tu et al., 2018[42]). Among the image features extracted from his study, 64 % aided in the differentiation of benign and malignant nodules and he showed evidence that under supervision of pathological diagnosis, with localized thin-section CT and radiomics feature extraction, physicians may find direct risk classification useful in diagnosing potential malignant nodules. Nasrullah et al. in 2019, proposed a deep learning-based model with multiple strategies including a three-dimensional (3D) customized mixed link network (CMixNet) for the detection and classification of pulmonary nodules. Initial detection was accelerated through Region-based Convolutional Neural Networks (R-CNN) based on the features from CMixNet and U-Net like encoder-decoder architecture, which was then classified with the aid of gradient boosting machine (GBM) (Nasrullah et al., 2019[27]). To have greater accuracy and to reduce false positives this was then co-related with biomarkers and physical symptoms to make an accurate diagnosis of the malignant nodules. Tran et al. introduced a deep learning method with 15-layer two-dimensional (2D) deep convolutional neural network (DCNN) architecture for automatic feature extraction and classification into nodule or non-nodule in CT scans (Tran et al., 2019[41]). Focal loss function was then used in the training process to improve the precision of the model and the results showed a specificity of 97.3 % , the sensitivity of 96.0 %, and an accuracy of 97.2 % on the Lung Image Database Consortium image collection (LIDC/IDRI) dataset extracted by the Lung Nodule Analysis 16 (LUNA16) challenge (Tran et al., 2019[41]). In 2019, Kailasam and Sathik (2019[18]) showed better effectiveness in terms of accuracy with DCNN and utilized it for feature extraction and hybridization as a combination of Histogram of Oriented Gradient (HOG), Extended Histogram of Oriented Gradients (ExHOG), Local Binary Pattern (LBP) and Convolutional Neural Network (CNN). Tao Sun et al. compared the support vector machine (SVM) classifier for lung cancer with other classifiers such as Boosting, Decision trees, k-nearest neighbor, LASSO regressions, neural networks, and random forests (Sun et al., 2013[38]). Compared to the reference methods, SVM classification was found to have an increased classification performance.

Characterization and classification of pulmonary nodules is an essential step in the screening and diagnosis of lung cancer. The management and prognosis of the disease are also severely impacted by this crucial step. Machine learning and computer-aided detection have revolutionized the characterization of lung nodules. Increased accuracy of classification was obtained through deep convolutional neural networks, even for a database with fewer images. Compared to typical algorithms, greater precision of characterization and classification of pulmonary nodules could be achieved through the proposed new models with FCM. A combination of the histogram and convolutional neural network was tried for classification and was found to have high accuracy. The novel classifiers developed, such as SVM, were also found to be superior to the reference classifiers. Regardless of nodule size, the deep learning-based computer-aided diagnosis has a higher detection rate compared to double reading. In the light of pathological diagnosis, integration of thin-section CT with machine learning and radiomics features allows direct risk classification. Co-relating three dimensional deep learning models with biomarkers and physical symptoms increases the accuracy of classification and diagnosis of malignant nodules. Two-dimensional DCNN has also been tried to extract features automatically with high sensitivity, specificity, and accuracy. To differentiate between the benign and malignant solitary pulmonary nodules of CT images, the AI-based schemes can be used as an auxiliary tool in the future. Deep convolutional neural networks in combination with several features have laid the foundation for the precise classification of lung nodules. Validation of these models and integration into routine practice allows analysis of a large amount of data on lung cancer and thereby reduce the workload and increase the efficiency of radiologists. This, in turn, can result in better patient care by early diagnosis, appropriate management, and favorable outcome.

## Limitations

A smaller sample size of studies and non-validated models are the main constraints limiting the research in this field. Constraints on computation and incomparable studies are also limiting in nature. Statistically significant results are not produced in some of the studies as the sample size is small. A systematically validated and confirmed model of convolutional neural network or machine learning is still not available for routine clinical use. The limited data access in the PubMed database and artificial intelligence being a field with frequent updates requires technologically sound analytical capacity. No mention of any adverse events related to the integration of AI and lung cancer screening has been seen in the available literature. The quality assessment of all the studies could not be carried out.

## Future Recommendations

Further research and studies are to be conducted and validation of the proposed models of convolutional neural networks has to be performed. Validation of the proposed models is required for the practical application of these in the screening procedure of lung cancer and thereby increasing the detection in earlier stages. More research and trials are to be conducted utilizing the technological advancements and the doctors have to take up the challenge to improvise and implement them.

## Conclusions

Artificial intelligence (AI) is one of the dynamic areas incorporated into imaging diagnostics, especially thoracic imaging, and we wanted to analyze the role and future potential of AI in the detection and classification of lung nodules for lung cancer screening. Deep learning and machine learning AI techniques promise a radical design for lung cancer screening in the future, due to their ability to manage a vast amount of data and automatically characterize pulmonary nodules with precision. Various combination models of convolutional neural networks, machine learning, handcrafted features, computer-aided diagnosis, spectrometry, genetic and molecular changes, provided better discrimination and evaluation of a greater proportion of lung nodules with higher sensitivity, specificity, and accuracy. Besides, the machine learning model combined with spectrometry developed protein marker panels for lung cancer detection and various other models enabling non-invasive breath tests or using public health information achieves greater detection accuracy. Unlike typical algorithms, deep convolutional neural networks increased the precision of classification and characterization of lung nodules with a higher detection rate. A combination of features is always superior and combining artificial intelligence with the performance of radiologists can develop a cost-effective and time saving efficient tool for lung cancer screening. However, validation of the models is essential in the future, for their implementation in the routine healthcare system so that it can be beneficial.

## Figures and Tables

**Table 1:**
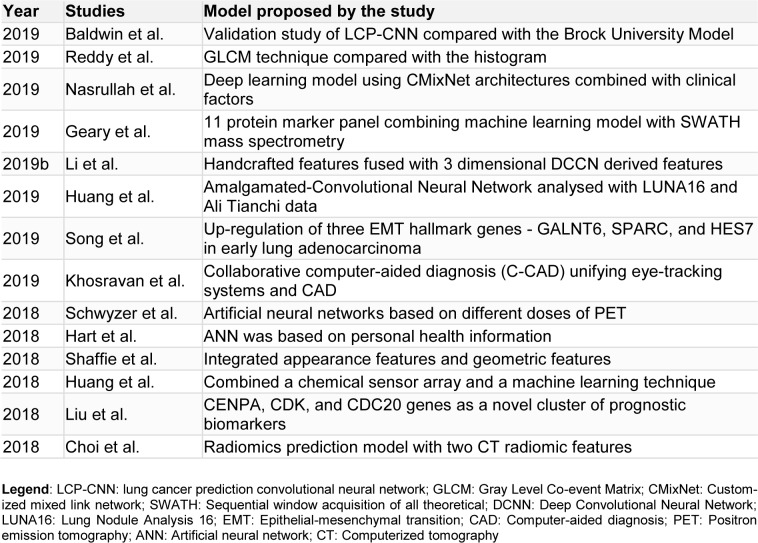
Summary of the models proposed for lung cancer screening
